# Association between rare variants in specific functional pathways and human neural tube defects multiple subphenotypes

**DOI:** 10.1186/s13064-020-00145-7

**Published:** 2020-07-10

**Authors:** Jizhen Zou, Fang Wang, Xueyan Yang, Hongyan Wang, Lee Niswander, Ting Zhang, Huili Li

**Affiliations:** 1 Beijing Municipal Key Laboratory of Child Development and Nutriomics, Capital Institute of Pediatrics, Beijing, 100020 China; 2 State Key Laboratory of Genetic Engineering and MOE Key Laboratory of Contemporary Anthropology, School of Life Sciences, Fudan University, Shanghai, 200438 China; 3 Obstetrics and Gynecology Hospital, Key Lab of Reproduction Regulation of NPFPC in SIPPR, Institute of Reproduction and Development, Fudan University, Shanghai, 200011 China; 4 Molecular, Cellular and Developmental Biology, University of Colorado Boulder, Boulder, Colorado 80309 USA

**Keywords:** Neural tube defect, Neural tube closure, Mutation, Spina bifida, Human

## Abstract

**Background:**

Neural tube defects (NTDs) are failure of neural tube closure, which includes multiple central nervous system phenotypes. More than 300 mouse mutant strains exhibits NTDs phenotypes and give us some clues to establish association between biological functions and subphenotypes. However, the knowledge about association in human remains still very poor.

**Methods:**

High throughput targeted genome DNA sequencing were performed on 280 neural tube closure-related genes in 355 NTDs cases and 225 ethnicity matched controls,

**Results:**

We explored that potential damaging rare variants in genes functioning in chromatin modification, apoptosis, retinoid metabolism and lipid metabolism are associated with human NTDs. Importantly, our data indicate that except for planar cell polarity pathway, craniorachischisis is also genetically related with chromatin modification and retinoid metabolism. Furthermore, single phenotype in cranial or spinal regions displays significant association with specific biological function, such as anencephaly is associated with potentially damaging rare variants in genes functioning in chromatin modification, encephalocele is associated with apoptosis, retinoid metabolism and one carbon metabolism, spina bifida aperta and spina bifida cystica are associated with apoptosis; lumbar sacral spina bifida aperta and spina bifida occulta are associated with lipid metabolism. By contrast, complex phenotypes in both cranial and spinal regions display association with various biological functions given the different phenotypes.

**Conclusions:**

Our study links genetic variant to subphenotypes of human NTDs and provides a preliminary but direct clue to investigate pathogenic mechanism for human NTDs.

## Background

Neural tube closure (NTC) is a highly orchestrated process wherein the neural plate bends to form the neural folds, which then meet and fuse to close the neural tube. In mammals, NTC initiates sequentially at different levels of the body axis [1]. Primary neurulation in mice initiates at the hindbrain/cervical boundary, called closure I, and then NTC proceeds by zippering together of the neural folds in a bidirectional manner into the hindbrain and along the spinal region [2]. Additional closure points are initiated at the rostral extremity of the forebrain (closure 3), and can also occur at the midbrain-forebrain boundary (closure 2). Fusion progresses along the spine, culminating in final closure at the posterior neuropore, at the level of the second sacral segment. Formation of the spinal cord at lower sacral and caudal levels is accomplished by a different process called “secondary” neurulation in which condensed mesenchyme hollows out to form a tube. Neuromesodermal progenitors biomechanically couple zippering point and drive caudal NTC [3]. In human embryos, neurulation events have been described that correspond to closure 1 and 3 in the mouse, whereas closure 2 may not occur in human embryos [4].

Perturbations of NTC can lead to neural tube defects (NTDs), a common and severe birth defect. Failure to close the neural tube in the cranial region (exencephaly or called anencephaly after degradation of the exposed neural tissue) leads to death before or at birth. Infants born with caudal NTDs (spina bifida) have increased risk of mortality, and those that survive often face life-long disabilities and neurological, cognitive, urologic, and gastrointestinal complications. A fundamental principle of neurulation is that the levels of the body axis that undergo primary neurulation are susceptible to “open” NTDs (for example, anencephaly, spina bifida aperta and craniorachischisis). By contrast, defective secondary neurulation leads to “closed” forms of spina bifida (also called “dysraphism” condition/spina bifida occulta), which represent the failure of the emerging spinal cord to become separated from other tissue derivatives of the tail bud. Failure of closure 1 leads to the most severe NTD, craniorachischisis, in which the neural tube is open throughout the midbrain, hindbrain and entire spinal region. If closure 1 is completed but closure of the cranial neural tube is incomplete, this leads to anencephaly. Failure of closure 3 is uncommon but, when present, yields split face with anencephaly. In the spinal region, failure of final closure at the posterior neuropore yields open spina bifida (Spina bifida aperta/Myelomeningocele), in which the upper limit can vary as to the axial level, depending on precisely when the progression of zippering becomes arrested.

In addition to the complex morphogenetic movements described above, numerous cellular and molecular functions must be tightly regulated to coordinate proliferation, differentiation, apoptosis, patterning and cell shape changes. Indeed, animal models have revealed over 300 genes that regulate the process of NTC, as disruption in the function of each gene can cause NTD. Animal models have also revealed how some of these genes contribute to NTC [1, 5, 6]. Narrowing and lengthening of the neural plate requires a process called convergent extension, wherein cell intercation mediolaterally in order to elongate the tissue along the rostrocaudal axis. Planar cell polarity (PCP) pathway is well documented to function in convergent extension [7–9] and mutations in PCP genes lead to failure to undergo closure 1, resulting in craniorachischisis [10]. Signaling through the BMP, Sonic hedgehog (Shh), FGF, Wnt pathways and cilia-related genes coordinate patterning of the neural tissue [11, 12]. Moreover, Shh signaling emanating from the notochord regulates dorsal lateral hinge point formation [13] and disruption of Shh signaling can lead to both spina bifida and exencephaly [1]. Apical constriction converts columnar cells into wedge-shaped cells, which involves cytoskeleton proteins such as Shroom3, Abl, and Mena [14]. Normal cytoskeletal function is required for cranial and spinal NTC and deficiency leads to exencephaly and spina bifida [1, 15]. Cell cycle is crucial for NTC and the balance between continued proliferation and neuronal differentiation may be critical for successful closure [1]. Live imaging has shown programmed cell death in cranial regions during neurulation [16] as well as dynamic cell behaviors and cell extensions during fusion [2, 15]. Prevention of neural fold fusion leads to hindbrain to forebrain exencephaly and thoracolumbosacral spina bifida [17–19]. Epigenetic modifiers, such as DNA methylation, chromatin modification and nucleosome assembly, contribute to proper closure of the mammalian neural tube [5]. Additionally, genes functioning in folate one carbon metabolism and glucose metabolism, as well as multiple vitamins and minerals are essential for NTC [20–22]. Despite the wealth of knowledge from animal models, there remains a large gap in knowledge relative to the association of these pathways with NTD phenotypes in humans.

Direct mutation screening of candidate genes has been carried out in cohorts of patients, largely using case-control association studies. NTDs are considered to be a complex disease with polygenic and multi-factorial etiology, and recent studies indicate that rare allele variation can have a large effect size of causation with respect to complex diseases [23]. Mutations in PCP pathway core genes *VANGL1* and *VANGL2* were explored first in craniorachischisis cases and then other NTD patients [24, 25], and the contribution of PCP gene mutations to human NTDs is well-established in several cohorts [26–28]. Another major emphasis has been on the evaluation of folate-related genes. A mutation screening study on rare variations within 31 folate-related genes in 480 NTD case-control population uncovered ethnic-specific risk signatures for spina bifida [29]. However, this has given inconsistent results between cohorts and populations, indicating that very few of the tested genes have a major causative effect [30]. We and others have screened a subset of retinoid-related genes in a range of NTD cases and found loss of function rare variants in retinoic acid degradative enzyme encoded gene *CYP26B1*, *CYP26A1* and retinoic acid receptor encoded genes [31–33].

To gain a broader understanding of the genetics of NTDs, in the present study we sequenced 280 NTC-related genes in 355 NTDs cases with multiple phenotypes and 225 ethnicity-matched controls in a high throughput manner. Our purpose was to identify rare mutations in a broad set of genes found to be critical for NTC in animal models and to then determine whether there is a relationship with human NTD phenotypes. This has allowed us to establish associations between molecular functions and some NTD sub-phenotypes in human.

## Subjects and methods

### Subjects

As described in our previous paper [33, 34], high-throughput DNA sequencing were performed on genomic DNA samples collected from 355 subjects with NTD in a Han Chinese population ranging in age from gestational week (GW) 12 to 10-years old and from multiple local hospitals in six provinces in China. Two hundred twenty-five ethnicity-matched controls were collected from non-medically related terminations and were free of any NTDs. The enrolled pregnant women were diagnosed by local clinicians using ultrasonography. Individuals with NTDs that had been assessed by clinical geneticists were enrolled and were placed into at least one of the following diagnostic groups: craniorachischisis, anencephaly, encephalocele, spina bifida (aperta, cystica, or occulta).

The study was approved by the Committee of Medical Ethics of the Capital Institute of Pediatrics (Beijing, China). Written informed consent was obtained from the parents. We carried out the study in accordance with The Code of Ethics of the World Medical Association (Declaration of Helsinki) for experiments involving humans and in accordance with the approved guidelines.

### Genomic DNA sequencing

Genomic DNA was extracted using a Truseq DNA Sample preparation kit (Illumina Inc., San Diego, CA), libraries constructed with Agilent Custom SureSelect Enrichment Kit, and run on an Agilent Custom enrichment array (Probe Code: BI426526171). Sequence reads were aligned to the UCSC human genome GRCh37/hg19 using BWA (v0.5.9) [35]. The average depth of coverage in the present sequencing was 22.5X. Based on cDNA sequence, nucleotide numbering uses + 1 as the A of the ATG translation initiation codon in the reference sequence, with the initiation codon as codon 1. Single-nucleotide variants (SNVs) were called using GATK (Samtools pileup (MAPQ30))(version 0.1.17) [36] and Varscan [37] (minimum coverage = 1, minimum alterative allele reads = 1, minimum variation frequency > 0.03) and short indels (insertions and deletions) were called using Varscan with a standard (minimum coverage = 2, minimum alternative allele reads = 2, minimum variation frequency > 0.1). Genotypes were called using Bayescall [38]. Variants were annotated with ANNOVAR [39]. Rare variants were filtered out using the dbSNP in NCBI, the 1000 Genomes Project, and the shared variants in NTDs cases and controls were also excluded.

## Results

### A wide range of NTD phenotypes in the current cohort

**Fig. 1:**
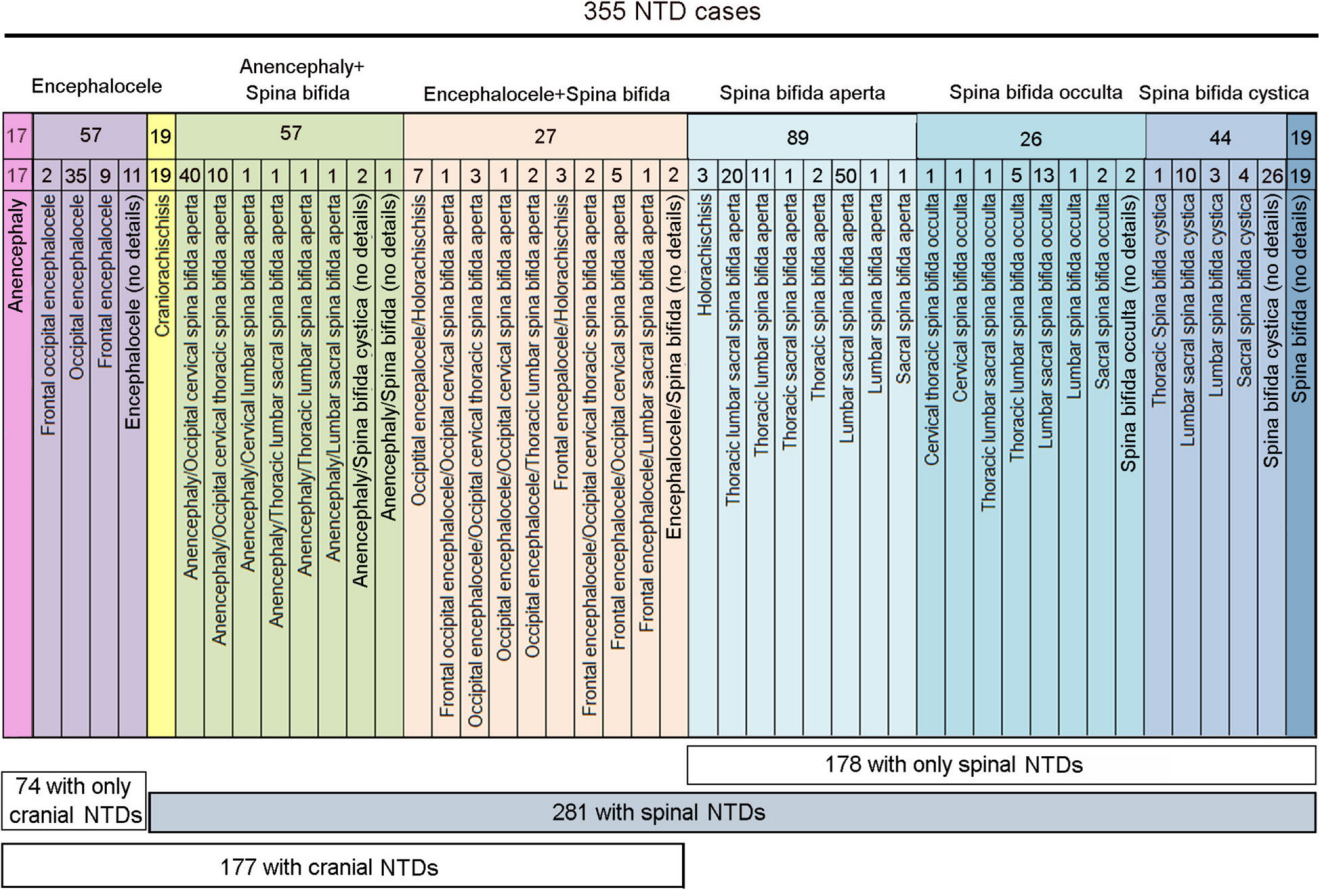
Category for neural tube defects cases in the present study. Phenotypes are arranged as order of rostral to caudal from left to right. The numbers in lower frames indicate summarized numbers of phenotypes

This study focuses on 355 NTD cases and 225 ethnicity-matched controls enrolled in China during 2005–2011. The NTD phenotypes were described as craniorachischisis (anencephaly continuous with exposed spinal cord), anencephaly (lack of brain and cranial vault subsequent to failure to close the cranial neural tube), encephalocele (meningeal sac, often containing brain tissue, protruding from the skull), spina bifida aperta (exposed spinal cord, usually called meningomyelocele), spina bifida cystica (spinal cord defect covered by meningeal sac), spina bifida occulta (skin-covered lesion involving two or more vertebrae, also called spina dysraphism) [40]. As shown in Fig. 1, 74 out of the 355 NTD cases showed cranial deficits only. This included 17 affected with anencephaly and 57 cases with encephalocele. Amongst encephalocele cases, 2 were located in the frontal and occipital regions, 35 were located in the occipital region, and 9 were in the frontal region. Of the remaining 11 encephalocele cases, detailed clinical information was not available (labeled encephalocele no details).

The number of cases with NTD limited to the spinal region was 178. The majority of these were spina bifida aperta (89 cases) which occurred in lumbar-sacral segments (50/89), thoracic-lumbar-sacral segments (20/89) and thoracic-lumbar segments (11/89) (Fig. 1). Of the 26 spina bifida occulta cases, the majority involved lumbar-sacral segments (13/26) and thoracic-lumbar segments (5/26). Many of spina bifida cystica cases did not have detailed information on the segments involved (26/44), hence we recruited 10 lumbar-sacral spina bifida cystica. Spina bifida cystica was not found in cervical segments (Fig. 1).

More complex phenotypes affecting both the cranial and spinal regions were also observed. This included 19 cases of craniorachischisis and 57 cases of anencephaly combined with spina bifida, the majority being anencephaly with upper segment spina bifida aperta (from thoracic to occipital). Four cases exhibited anencephaly and lower level spina bifida aperta encompassing the lumbar or sacral segments (Fig. 1). The remaining 27 cases were encephalocele combined with spina bifida aperta (Fig. 1; in 2 cases there was no detailed clinical information).

A summary of the phenotypes in the current cohort is 74 cases with only cranial malformations, 178 cases with only spinal phenotypes, and 103 cases with complex phenotypes in both cranial and spinal regions. Viewing all cases together, there are 177 with cranial NTDs and 281 with spinal NTDs (Fig. 1).

### High throughout targeted sequencing of neural tube closure (NTC)-related genes

To gain an understanding of the genetic contribution to a broad set of NTD phenotypes, we undertook a population-based case-control mutation screening study to reveal potential disease-causative rare variants by sequencing a selected set of 280 genes implicated in NTC from human and animal studies [1, 5, 6, 30, 40]. These 280 genes encompassed more than twenty-one signaling pathways and key developmental pathways (upplementary Table 1). For these 280 genes we sequenced the coding regions (CDS), splice junctions, and 2000 bases upstream of the genes for a total of 4.38 Mb of the human genome. The CDS length sequenced for each pathway is shown in Supplementary Table 1. We applied criteria in calling cohort-specific variants by eliminating from further analysis shared rare variants found in both NTD and control samples. SIFT and Polyphen2 were used to identify putatively damaging SNVs [41, 42]. All missense mutations, together with splicing mutations, frameshift mutations or nonsense mutations were defined as putatively damaging rare variants (PDRVs) in the present study. By these criteria we identified NTDs case-specific 791 PDRVs in 213 genes and 387 common PDRVs in 150 genes only in controls, all PDRVs were heterozygous.

### PDRVs in the pathways of chromatin modification, apoptosis, retinoid metabolism and lipid metabolism are enriched in NTDs

**Fig. 2:**
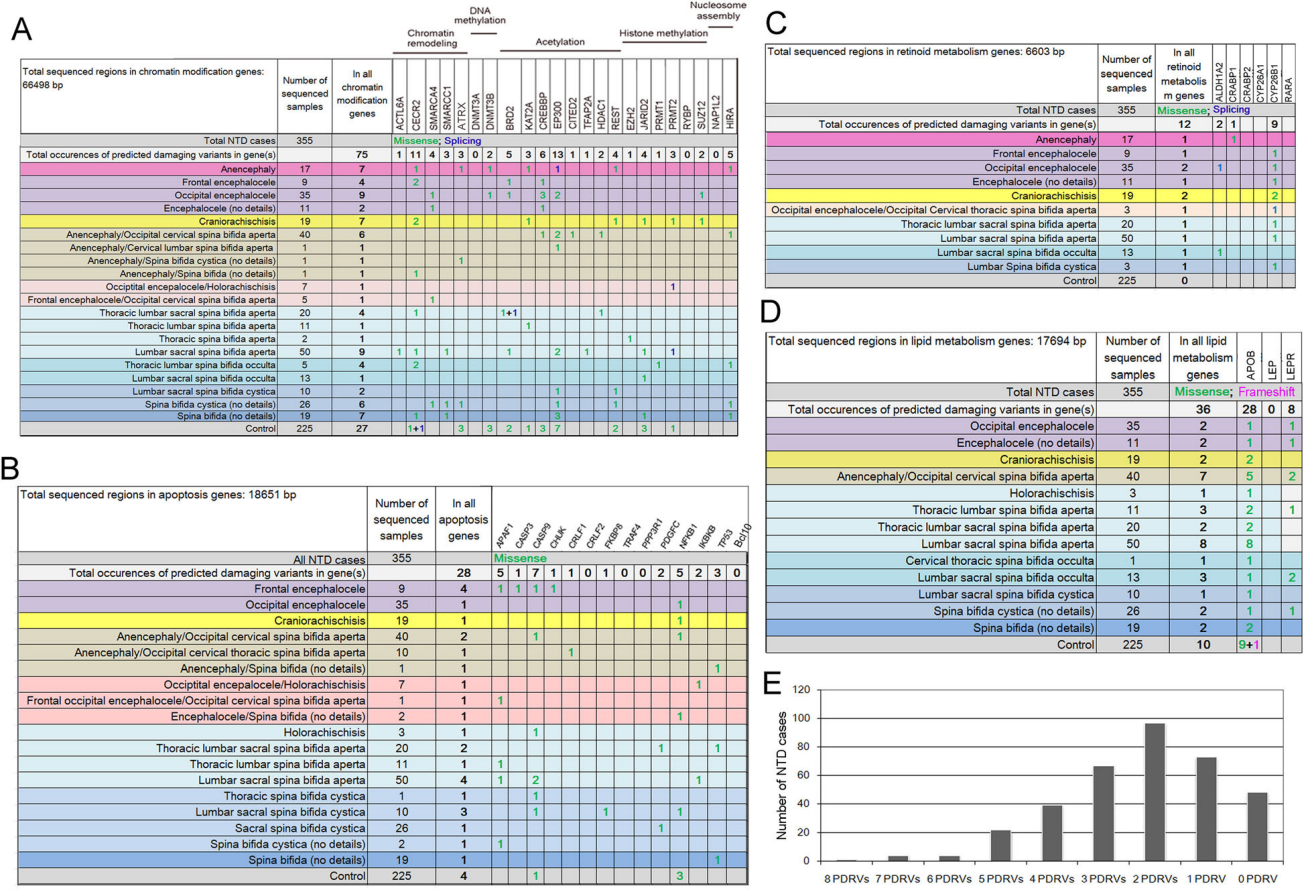
Four pathways significantly associated with human neural tube defects. In the present study, PDRVs in genes functioning in four pathways have been explored being associated with the current NTD cohort, they are (**a**) chromatin modification; (**b**) apoptosis; (**c**) retinoid metabolism related genes; and (**d**) lipid metabolism. The occurrences of missense variants are shown as green font; of splicing variants are shown as blue; of frameshift variants are shown as purple. The background color of different phenotypes are same as in Fig. 1. (**e**) Polygenic or digenic genetic PDRVs were observed in the present NTD cohort. The columns represent the number of NTD cases who carries certain numbers of PDRVs in different genes

To explore the possible genetic contribution of each signaling pathway to human NTDs, we first separately analyzed the number of PDRVs in NTDs and controls within each pathway. The data indicate that the genes which function in chromatin modification, apoptosis, retinoid-related and lipid metabolism harbor significantly more NTD-specific PDRVs (Fig. 2). Regarding genes that function in chromatin modification, we found 75 PDRVs in NTD cases which was significantly more than in controls (75/355 in NTDs *vs.* 27/225 in controls; *P* = 0.019; two-sided Fisher’s exact test) (Fig. 2a). For genes that function more specifically in chromatin remodeling (*ACTL6A, CECR2, SMARCA4, SMARCC1* and *ATRX*) there were 22 PDRVs in 355 NTD cases, which was slightly but significantly more than in controls (5/225; *P* = 0.041, two-sided Fisher’s exact test).

Defective apoptosis can lead to cranial NTD in mouse [16]. Here we analyzed 14 genes implicated in the apoptosis pathway and found 28 PDRVs in NTD cases, significantly enriched relative to controls (28/355 vs. 4/225, *P* = 0.0023, two-sided Fisher’s exact test) (Fig. 2b). Interestingly, the intrinsic pro-apoptotic genes implicated in exencephaly in mice *TP53* [43], *APAF1* [44], *CASP9* [45] and *CASP3* [46] harbored 16 PDRVs in human NTD cases, considerably increased relative to controls (16/355 vs. 1/225, *P* = 0.0044, two-sided Fisher’s exact test) (Fig. 2b). By contrast, we did not find a PDRV in the anti-apoptotic gene *BCL10* in our cohort (Fig. 2b), although its mutant show exencephaly in mice [47].

Retinoic acid is a small lipophilic molecule derived from vitamin A via the retinoid metabolism pathway and gene mutations in this pathway cause NTD phenotypes in mice [48]. As we previously reported [33], in six retinoid metabolism genes we found 12 occurrences of PDRVs in NTD cases versus 0/225 in controls (*P* = 0.0046, two-sided Fisher’s exact test) (Fig. 2c) and PDRVs were predominantly in the *CYP26B1* gene (9/12), which acts to degrade retinoic acid. In the three genes sequenced in the lipid metabolism pathway, there were significantly more PDRVs in NTD cases (36/355 in NTDs *vs.* 10/225 in controls, *P* = 0.025) (Fig. 2d). Of note, a frameshift variant in the *APOB* gene was found in control (NM_000384: exon26:c.10373delT; p.M3458fs) but overall the *APOB* gene harbored more PDRVs in NTD cases (28/355) versus controls (10/225). PDRVs in the *LEPR* gene were only found in NTD cases (8/355 *vs. *0/225, *P* = 0.026, Fig. 2d). In total, in the current NTD cohort, polygenic PDRVs were carried by 137 cases, while 97 cases were digenic, by contrast, no PDRV in the targeted genes was found in 48 cases (Fig. 2e). These confirm polygenic or digenic genetic features for human NTDs, however, it is also necessary to extend the target list in the future.

In summary, evaluation of our current set of 280 genes and 21 pathways showed that four signaling pathways were significantly enriched for NTD-specific PDRVs. Furthermore, PDRVs in genes of these four pathways were associated with 34% (120/355) of NTD cases. These data suggest the importance of PDRVs in these pathways in the pathogenesis of NTDs and highlight these biological functions as critical for human NTC.

### Craniorachischisis is associated with PDRVs in PCP signaling and new pathways of chromatin modification and retinoid metabolism

**Fig. 3:**
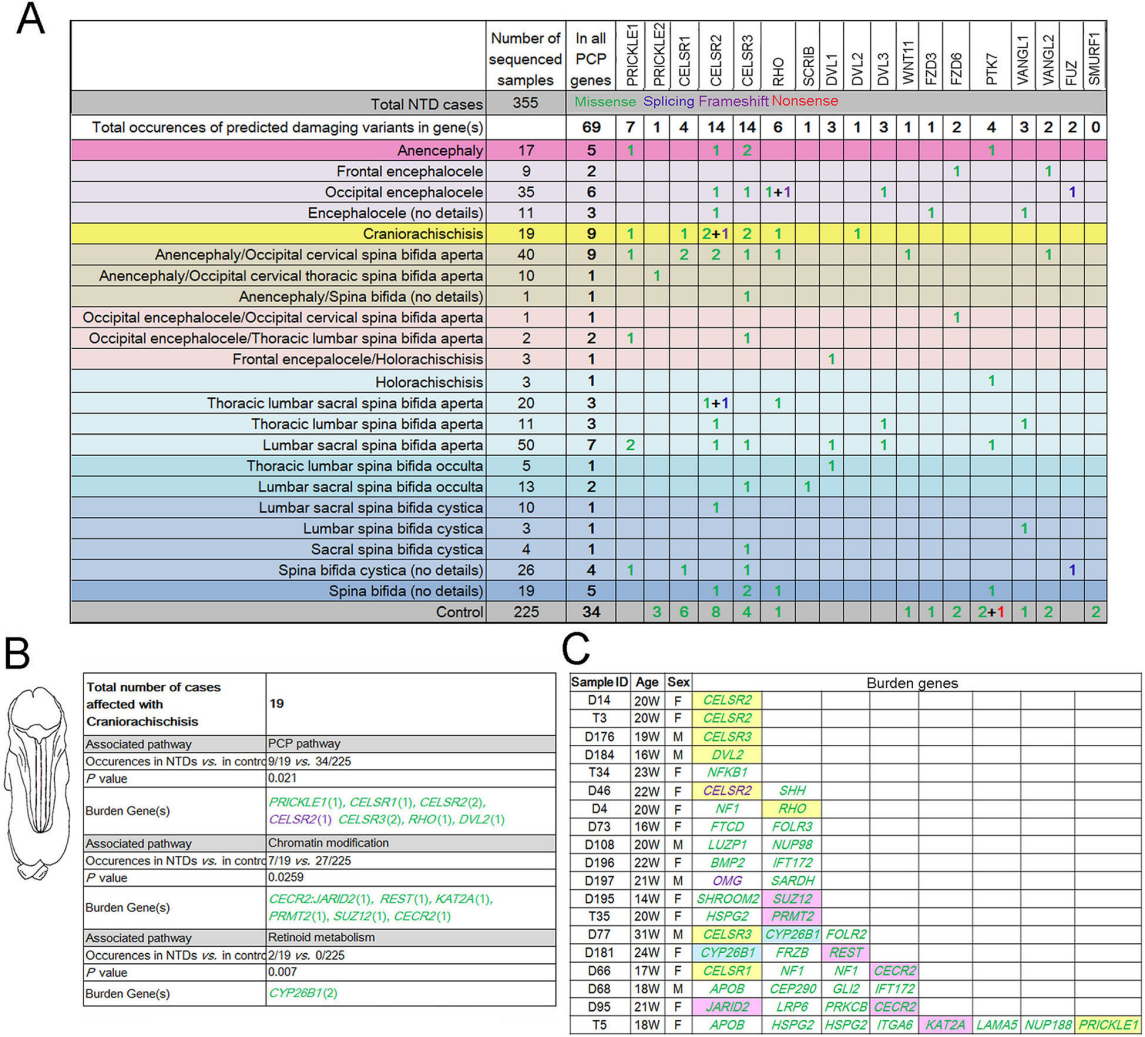
Planar cell polarity pathways and craniorachischisis. In the present cohort PDRVs occur in multiple NTD phenotypes but are significantly associated with craniorachischisis (**a**). The background color of different phenotypes are same as in Fig. 1. Interestingly, except for planar cell polarity pathway, craniorachischisis phenotype is statistically associated with PDRVs in chromatin modification and retinoid metabolism genes (**b**), fisher exact test was used to calculated *P* value, and numbers in brackets means occurrences of PDRVs in each gene. In (**c**) we list all 19 craniorachischisis in the present study and the PDRVs we found in these cases. Yellow background means genes in planar cell polarity pathway; purple background represents genes functioning in chromatin modification; sky blue background represents retinoid metabolism related genes. In all panels, numbers or gene symbols in green represent missense PDRVs occurred; in blue represent splicing PDRVs; in purple represents frameshift PDRVs and in red represents nonsense PDRVs

We next asked whether we could discern possible relationships between more specific NTD phenotypes and variants in molecular pathways. This was possible as our present cohort encompasses a range of NTD sub-phenotypes including cranial, caudal, and complex NTD as shown in Fig. 1. We started with an analysis of craniorachischisis, the most severe NTD phenotype and which has been associated with defects in genes within the PCP pathway in both mice [5] and human [49]. Our sequencing study encompassed 18 PCP genes, including the core PCP genes *VANGL1/2*, *DVL1/2/3, CELSR1* and *PRICKLE1/2* [50]. The results showed that there were 69 PDRVs in PCP genes in the total cohort of 355 NTD cases versus 34 PDRVs in 225 controls, thus not reaching statistical significance when viewed across the range of NTDs cases (Fig. 3a). However, as expected, PDRVs in PCP genes were significantly associated with craniorachischisis (9/19 in craniorachischisis cases *vs.* 34 in all 225 controls; *P* = 0.0210; two-sided Fisher’s exact test, Fig. 3b). Within this smaller cohort of 9 craniorachischisis cases, there were 9 different occurrences of PDRVs in PCP pathway genes (yellow background in Fig. 3c highlights these 9 cases). Thus, our data support previous findings that the PCP pathway genetically contributes to human craniorachischisis. It is of note that some craniorachischisis do not carry PDRVs in the set of PCP pathway genes sequenced, either suggesting the presence of variants in untranslated regions of the sequenced PCP genes or in other PCP genes that were not sequenced or alternative mechanism(s) underlying this pathology. In support of the latter possibility, we found a relationship between PDRVs in genes involved in chromatin modification and retinoid metabolism that were associated with the craniorachischisis phenotype (Fig. 3b, pink and blue highlight in Fig. 3c). Interestingly, the degradation enzyme for retinoic acid encoded by the *CYP26B1* gene harbors significant number of PDRVs in craniorachischisis cases (2/19 in craniorachischisis *vs.* 0/225 in all controls; *P* = 0.007; Fig. 3b and c). Therefore, our sequencing of even a limited number of NTD genes reveals new candidate genes related to chromatin modification and retinoid metabolism associated with craniorachischisis. Furthermore, our sequencing uncovered additional genes and pathways (Fig. 3c) that may play causal roles and are worthy of consideration in other cases of this most severe NTD, craniorachischisis.

### Cranial NTD phenotypes are associated with PDRVs in chromatin modification, retinoid and glucose metabolism, apoptosis and one-carbon metabolism

**Fig. 4:**
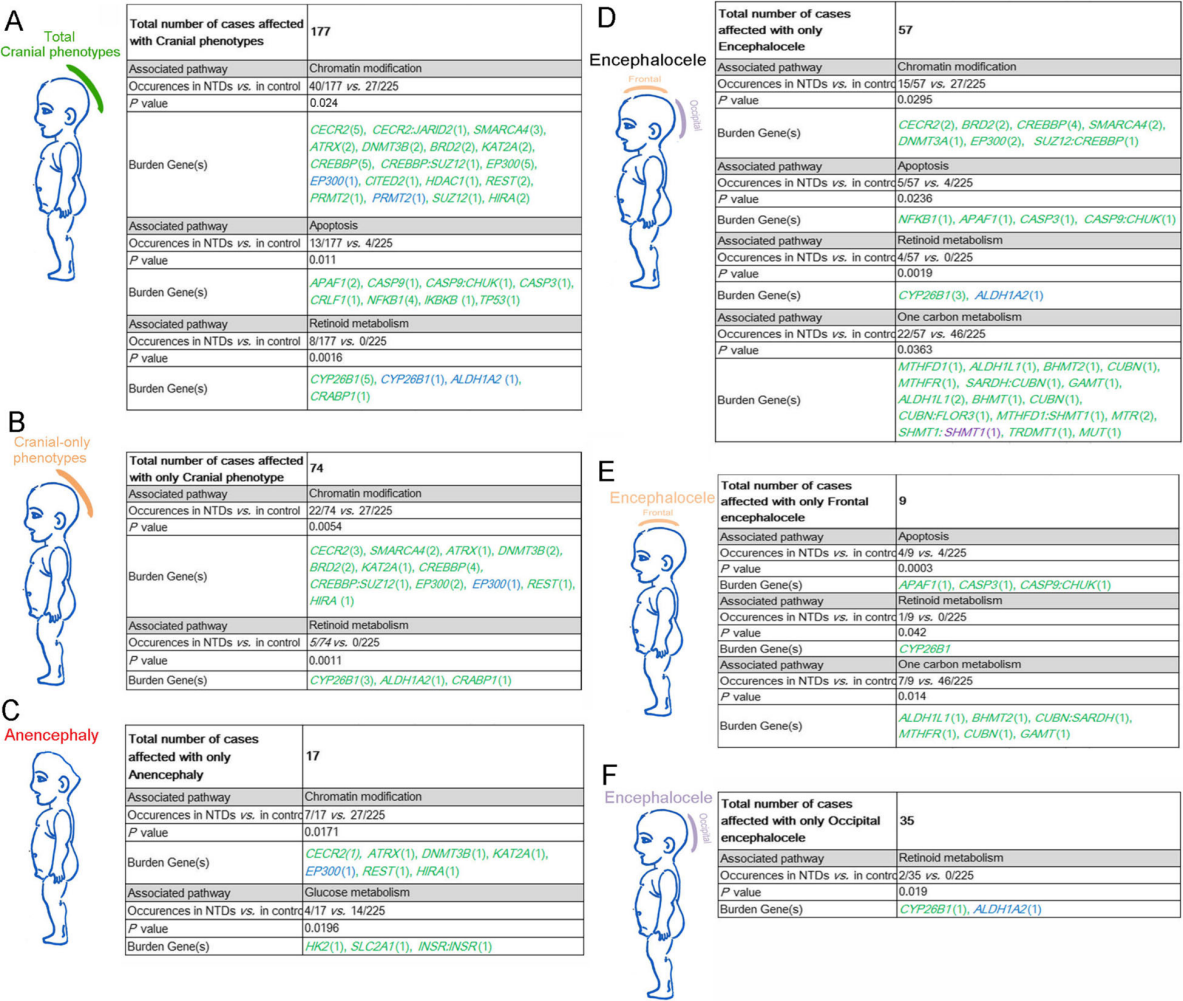
Summary on PDRVs burden genes related functional pathways in cranial NTDs. (**a**) Summary of PDRVs in all 177 NTDs cases who display NTDs phenotype in cranial regions with or without spinal regions. (**b**) Summary in cases who have only cranial NTD phenotypes. (**c**-**f**) show summaries of PDRVs burden genes and their functional pathways in cases affected with only anencephaly (**c**); only encephalocele (**d**); only frontal encephalocele (**e**); only occipital encephalocele (**f**). Numbers in brackets means occurrences of PDRVs in each gene, numbers or gene symbols in green represent missense PDRVs; in blue represent splicing PDRVs; in purple represents frameshift PDRVs. Gene symbols linking with colon represents two concurrent PDRVs in case

We next addressed whether particular pathways may be more closely associated with cranial NTD sub-phenotypes. Statistical analysis of the 177 NTD cases that include a cranial phenotype (Fig. 1) indicated that PDRVs in genes in chromatin modification, apoptosis and retinoid metabolism were associated with prevalence of cranial NTD phenotypes with or without spinal NTD phenotypes (*P* = 0.024; *P* = 0.011 and *P* = 0.0016, respectively; Fig. 4a). Regarding cranial-only NTD phenotypes, PDRVs were enriched in genes involved in chromatin modification and retinoid metabolism (*P* = 0.0054 and *P* = 0.0011; Fig. 4b). In 17 cases affected with only anencephaly (Fig. 4c), PDRVs were enriched in chromatin modification genes as well as in glucose metabolism genes, particularly the case D102 carries two different PDRVs in the *INSR* gene (*INSR*: NM_000208: c.1882C > G:p.P628A and c.4028G > A:p.R1343Q).

In all 57 encephalocele cases, PDRVs in genes in chromatin modification, apoptosis, retinoid metabolism and one carbon metabolism were found significantly associated with the phenotype prevalence (Fig. 4d). Interestingly, three pathways were related to encephalocele in frontal regions (Fig. 4e), whereas for occipital encephalocele the PDRVs were statistically accumulated only in genes functioning in retinoid metabolism (Fig. 4f), correlating with a role for retinoid signaling in hindbrain patterning [51]. In general, it appears that PDRVs in genes in chromatin modification can contribute to diverse cranial phenotypes with or without spinal phenotypes, including anencephaly and encephalocele. In contrast, PDRVs in genes in retinoid metabolism are associated with all types of encephalocele, but not anencephaly, at least in our cohort of patients. Finally, PDRVs in genes in apoptosis and one carbon metabolism are significantly associated with frontal encephalocele or encephalocele only (Fig. 4d and e).

### Spina bifida phenotypes are associated with PDRVs in apoptosis, retinoid and lipid metabolism, and cytoskeleton genes

**Fig. 5:**
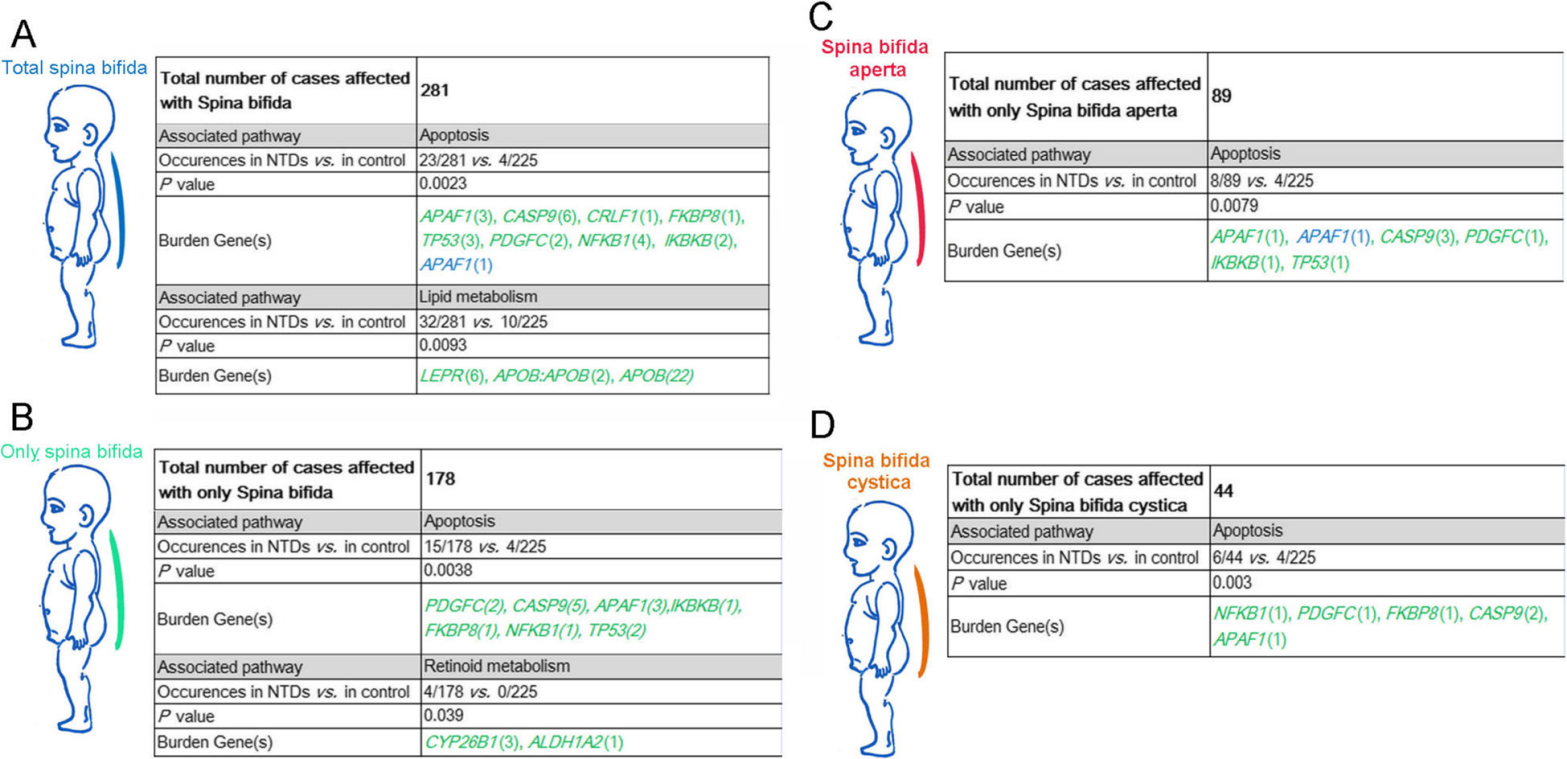
Summary on PDRVs burden genes related functional pathways in spinal NTDs. Summary in cases affected with spinal NTD phenotype whatever who are with or without cranial NTD (**a**), or affected with only spinal NTD phenotypes (**b**), or with only spina bifida aperta (**c**), or with spina bifida cystica (**d**). Numbers in brackets means occurrences of PDRVs in each gene, numbers or gene symbols in green represent missense PDRVs; in blue represent splicing PDRVs; in purple represents frameshift PDRVs. Gene symbols linking with colon represents two concurrent PDRVs in case

Spina bifida remains a common NTD phenotype worldwide and we enrolled a total of 281 cases with spina bifida, of which 178 had only spinal NTDs (Fig. 1). When viewed as the entire cohort of 281 spina bifida with or without cranial phenotype, there was significant enrichment for PDRVs in apoptotic pathway genes (23/281 *vs. *4/225 controls; *P* = 0.0023, two-sided Fisher’s exact test) and lipid metabolism genes (32/281 *vs. *10/225 controls; *P* = 0.0093, two-sided Fisher’s exact test) (Fig. 5a). However, when the 178 cases with only spinal NTDs were considered, the association with lipid metabolism genes was not significant. Instead, these 178 cases were significantly associated with PDRVs in apoptosis and retinoid metabolism genes (15/178 and 4/178, respectively in spina bifida cases *vs. *4/225 and 0/225 in controls, Fig. 5b). In addition, PDRVs in apoptosis genes are related to spina bifida aperta and spina bifida cystica (*P* = 0.0079 and *P* = 0.030, Fig. 5c and d). Taken together, PDRVs in apoptosis genes seem to be highly associated with cases of spina bifida aperta and spina bifida cystica.

**Fig. 6:**
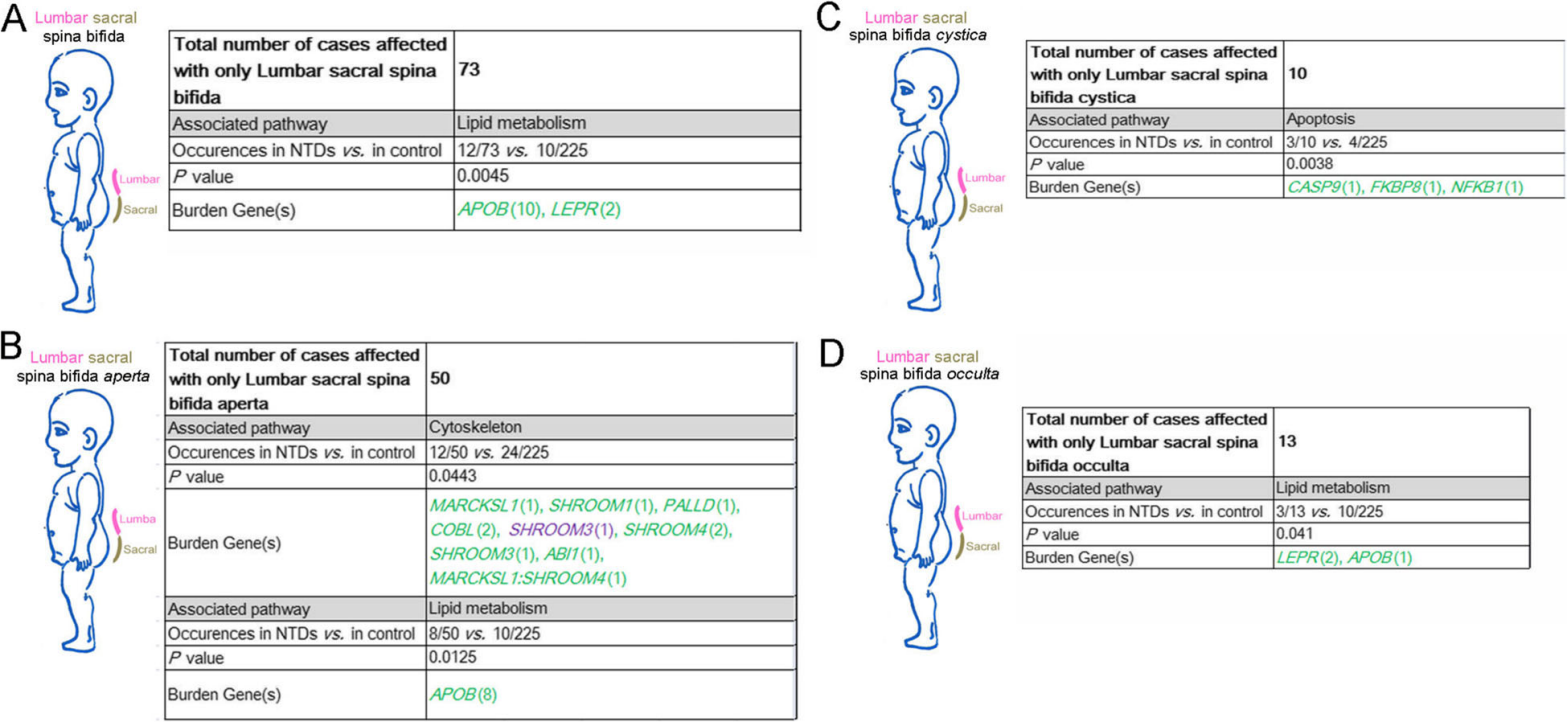
Summary on PDRVs burden genes related functional pathways in lumbosacral spina bifida. Summary in cases affected with only lumbar sacral spina bifida (**a**), or affected with only spinal NTD phenotypes (**b**), or with only spina bifida aperta (**c**), or with spina bifida cystica (**d**). Numbers in brackets means occurrences of PDRVs in each gene, numbers or gene symbols in green represent missense PDRVs; in blue represent splicing PDRVs; in purple represents frameshift PDRVs. Gene symbols linking with colon represents two concurrent PDRVs in case

Because we had a large cohort of spina bifida cases that varied in position of the affected segments as well as severity, we next evaluated our data within specific caudal NTD sub-phenotypes. Lumbar sacral spina bifida is thought to result from failure of primary neurulation at the caudal end and failure of secondary neurulation [1]. Our present cohort included 73 cases with only lumbar sacral spina bifida and these showed an association only with lipid metabolism genes (Fig. 6a). PDRVs in the *APOB* gene occurred in a significant number of lumbar sacral spina bifida cases (10/73 *vs.* 10/225 controls, *P* = 0.018, two-sided Fisher’s exact test), although *Apob* knockout mice exhibit only exencephaly [52]. We then separated the lumbar sacral spina bifida cases into spina bifida aperta, spina bifida cystica, and spina bifida occulta. Lumbar sacral spina bifida aperta is the most widespread NTD phenotype in human. In 50 cases, we found PDRVs in genes functioning in cytoskeleton and lipid metabolism (8 PDRVs in the *APOB* gene) were significantly enriched (*P* = 0.044 and 0.012, respectively; Fig. 6b). The regulation of the actin cytoskeleton is critical in murine NTC [5] and we found PDRVs in many genes that control the actin cytoskeleton including the SHROOM family of proteins. Our data identified a frameshift variant in the *SHROOM3* gene in case D183 (NM_020859:c.1782delC:p.N594fs; indicated in orange font in Fig. 6b) and another case (TJ-QS39) which carries a *SHROOM4* NM_020717:c.2011G > C: p.A671P variant and a *MARCKSL1*:*NM_023009*:c.226G > A: p.A76T variant (Fig. 6b).

In the 10 lumbar sacral spina bifida cystica cases, we found PDRVs in 3 apoptotic genes (*P* = 0.0038; Fig. 6c). For the 13 lumbar sacral spina bifida occulta cases, PDRVs in genes in lipid metabolism were weakly correlated (*P* = 0.041, Fig. 6d). Although the number of NTD sub-phenotype cases is too few to draw strong inferences, it is interesting that lumbar sacral spina bifida occulta was related to PDVRs in *LEPR* gene (2/13 *vs.* 0/225, *P* = 0.0037), whereas PDRVs in *LEPR* were not associated with lumbar sacral spina bifida aperta.

Within our enrolled cases there were a few other spinal sub-phenotypes encompassing other segments. In eleven cases affected by thoracic lumbar spina bifida aperta there were 3 PDRVs in the lipid metabolism genes *LEPR* and *APOB* (*P* = 0.029; Fig. S1A). Interestingly, the case D126 carried two *APOB* PDRVs (*APOB*:NM_000384:c.11230C > G: p.L3744V and c.7777A > G: p.I2593V). Within our two cases of thoracic spina bifida aperta-only, one case carried two PDRVs in genes involved in nuclear pore complex function (*NUP98*:NM_139132:c.4837C > T: p.R1613C and c.5026C > A: p.H1676N) (Fig. S1B). Our five cases of thoracic lumbar spina bifida occulta harbored PDRVs in genes in chromatin modification (4/5* vs.* 27/225 controls) and neural development (3/5 *vs.* 12/225 controls; *P* = 0.013 and *P* = 0.008, respectively; Fig. S1C). Three cases of lumbar spina bifida cystica revealed one PDRV in the retinoid metabolism gene *CYP26B1* (1/3 *vs.* 0/225, *P* = 0.017; Fig. S1D). In the one case of thoracic spina bifida cystica there was a PDRV in apoptosis gene *CASP9* (*P* = 0.042; Fig. S1E).

### Complex NTDs affecting both cranial and spinal regions are associated with PDRVs in a range of functional pathways

**Fig. 7:**
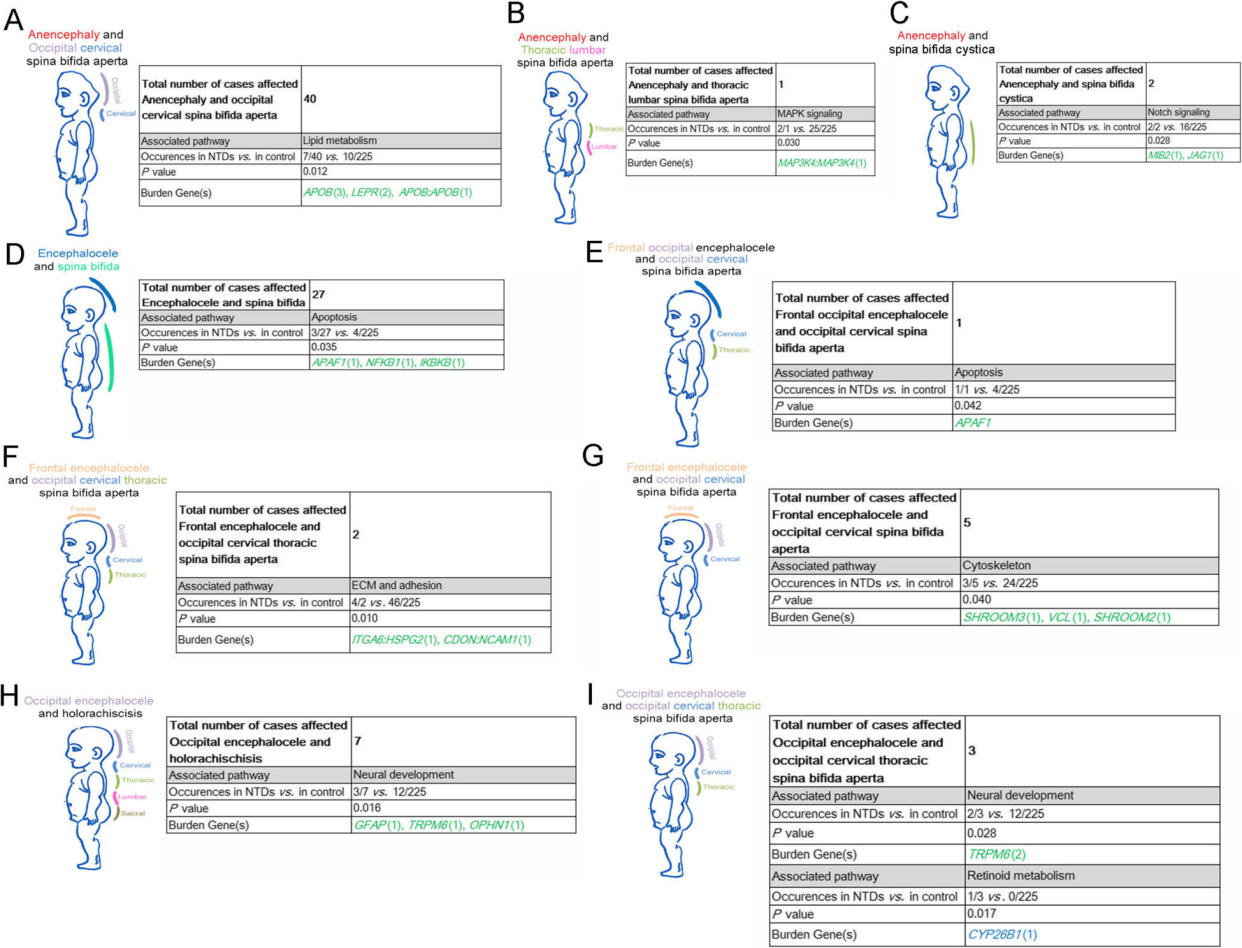
Summary on PDRVs burden genes related functional pathways in cranial and spinal complex phenotypes**.** Summary of PDRVs burden genes in cases affected with anencephaly and spinal NTD phenotypes (**a**-**c**), or encephalocele and spinal NTD phenotypes (**d**-**i**). Numbers in brackets means occurrences of PDRVs in each gene, numbers or gene symbols in green represent missense PDRVs; in blue represent splicing PDRVs; in purple represents frameshift PDRVs. Gene symbols linking with colon represents two concurrent PDRVs in case

Our present cohort also encompasses complex phenotypes which affect both cranial and spinal regions. In the 40 cases with anencephaly and occipital cervical spina bifida aperta, the sequencing data revealed 7 PDRVs in lipid metabolism genes (*P* = 0.011, Fig. 7a). This included the case D98 which carries two heterozygous *APOB* missense variants (*APOB:* NM_000384 c.11230C > G: p.L3744V and c.7777A > G: p.I2593V)(Fig. 7a). Interestingly, we had one case with anencephaly and thoracic lumbar spina bifida aperta (the neural tube was closed in cervical region), who carried two missense mutations in the *MAP3K4* gene involved in MAPK signaling (*MAP3K4*: NM_005922: c.877G > A: p.D293N and c.2711C > G: p.T904S)(Fig. 7b). In mouse Map3k4 expresses along the edges of neural folds and the mutant mice display exencephaly from forebrain to hindbrain, curly tail and lower spina bifida phenotypes or combination of exencephaly and spina bifida/curly tail [53, 54]. In two cases with anencephaly and spina bifida cystica, missense mutations were found In two Notch signaling pathway genes; one in the E3 ubiquitin-protein ligase *MIB2*, which positively regulates the Delta-mediated Notch signaling, and the other in *JAG1*, a ligand for multiple Notch receptors (2/2 *vs. *16/225 controls, *P* = 0.028; Fig. 7c). In 27 cases of encephalocele and spina bifida there were 3 PDRVs in apoptotic genes (*P* = 0.035; Fig. 7d). In an unique case with frontal occipital encephalocele and occipital cervical spina bifida aperta, there was a PDRV in the pro-apoptotic gene *APAF1* (*APAF1*:NM_181868:c.1004C > G: p.P335R; *P* = 0.042; Fig. 7e). Notably, in 2 cases of frontal encephalocele and occipital cervical thoracic spina bifida apertas, there were 4 PDRVs in four genes functioning in ECM and adhesion (4/2 *vs.* 46/225 controls; *P* = 0.010; Fig. 7f). This included concurrent PDRVs in integrin alpha6 encoded gene *ITGA6* and a basement membrane protein heparin sulfate proteoglycan 2 encoded gene *HSPG2* in one individual; and concurrent PDRVs in two cell adhesion genes *NCAM1* and *CDON* in another individual. This suggests a possible synergistic effect of ECM and adhesion on the closure events disrupted in cases of frontal encephalocele and occipital cervical thoracic spina bifida aperta. In 5 cases of frontal encephalocele and occipital cervical spina bifida aperta, we observed 3 PDRVs in the cytoskeleton genes *SHROOM2, SHROOM3*, and *VCL* encoding VINCULLIN (*P* = 0.040; Fig. 7g). There was a relationship to PDRVs in neural development genes with the complex phenotypes of occipital encephalocele and spina bifida represented by occipital encephalocele and holorachischisis (spina bifida aperta in all spinal regions, 3 PDRVs in 7 cases vs. 12/225) and occipital encephalocele and occipital, cervical and thoracic spina bifida aperta (2/3 cases vs. 12/225) (*P* = 0.016 and *P* = 0.028, respectively; Fig. 7h and i). *TRPM6*, encodes a channel protein crucial for magnesium homeostasis and mutations in *Trpm6* in mice result in exencephaly and spina bifida occulta [55]. We found 2 PDRVs in *TRPM6* in two cases of occipital encephalocele and occipital, cervical and thoracic spina bifida aperta and 1 PDRV in one case of occipital encephalocele and holorachischisis (*P* = 0.0004 and *P* = 0.0343; respectively, Fig. 7h and i).

## Discussion

The association of craniorachischisis and PCP gene variants should be highlighted in human NTD genetics [34, 49, 50]. In the present study, our data indicated that in addition to PCP genes, variants in retinoic acid related gene *CYP26B1* and chromatin modification genes are associated with craniorachischisis prevalence. This suggests that genetic variants in PCP genes are not unique causative factors for craniorachischisis in human and reveals new genes for consideration for this severe NTD. Indeed, mouse embryos deficient for glycine decarboxylase (*Gldc*) exhibit craniorachischisis as well [22]. The CYP26 proteins along with retinaldehyde dehydrogenase control the regional levels of retinoic acid. Cyp26b1, together with Cyp26a1 and Cyp26c1, corrects Nodal expression during gastrulation, when retinaldehyde dehydrogenase is not expressed [56]; mice lacking all three CYP26 genes manifest duplication of neural tube which is more pronounced than *Cyp26a−/c-* double mutant mice [56], emphasizing the crucial role of Cyp26b1. Chromatin modification genes are necessary for NTC, although mouse embryos carrying mutations in these genes mainly exhibit exencephaly [1]. Nonetheless, multiple chromatin modification genes such as *Dnmt3b* and *Cecr2* show expression patterns distributed at the leading edge of the neural folds along the rostrocaudal axis during neurulation [57, 58]. *Kat2a* is also expressed widely along the cranial and spinal neural tube [59]. *Jarid2*, a regulator of histone methyltransferases complex, is expressed at the midbrain-hindbrain boundary and distributed in spinal regions as well [60]. These suggest that these molecules potentially play roles in both spinal and cranial regions. Moreover, in our present results, all variants in chromatin modification genes and *CYP26B1* gene are concurrent with other variants in neural tube-related genes (Fig. 3c), so we hypothesize that the craniorachischisis phenotype is also resulted from the synergistic effect of NTC-related polygenic functional variants.

Rare variants in chromatin modification genes, apoptosis genes and retinoid metabolism genes significantly contribute to cranial NTD phenotypes, including anencephaly and encephalocele (Fig. 4), further emphasizing the importance of these signaling pathways in cranial neurulation. The morphology of the elevating neural folds differs between cranial and spinal regions in mammals. In the mouse embryonic midbrain, the neural folds are initially bi-convex with their tips orientated away from the midline. Then, dorsolateral bending occurs, generating bi-concave neural folds and orienting the tips towards the midline for fusion [50, 61]. In addition, the emigration of cranial neural crest may enable dorsolateral bending [4]. In contrast, spinal region does not exhibit a bi-convex elevation phase and the neural folds remain straight except for focal bending sites at the midline and dorsolaterally; and neural crest emigration in the spinal region does not begin until several hours after NTC is complete [62]. In addition, during cranial neurulation, a functional actin cytoskeleton, emigration of the cranial neural crest, spatio-temporally regulated apoptosis, and a balance between cell proliferation and the onset of neuronal differentiation are all required for normal dorsolateral bending [50]. Moreover, closure of the cranial neural tube is essential not only for maintenance of brain development but also for initial formation of much of the skull, with contributions from both cranial mesenchyme and cranial neural crest [4, 63]. Coordinate regulation of transcriptional networks by epigenetic regulators is absolutely crucial for proper cranial NTC [5].

In our present NTD cohort, variants in one carbon metabolism are significantly associated with encephalocele, in particular frontal encephalocele (Fig. 4d and e). A previous study of 239 newborns with spina bifida and 241 non-malformed controls reported a Hispanic genetic risk profile pointing to alterations in genes functioning in purine biosynthesis, whereas that in non-Hispanic whites implicated in homocysteine metabolism [29]. In glycine cleavage system genes, rare variants are associated with multiple phenotypes including spina bifida and anencephaly [22, 64]. Therefore, except for a possible causation of different ethnicities, association of one carbon metabolism to NTD subphenotypes is still to be determined in a larger number of populations.

Variants in the lipid metabolism genes *APOB* and *LEPR* appear to contribute to lumbar sacral spina bifida in the present study (Fig. 6). NTC in lumbar sacral regions is attributed to secondary neurulation. Therefore, our data provides clues that lipid metabolism is essential for this process in human. *APOB* encoded apolipoproteins B is a major structural component of very low density lipoproteins, intermediate density lipoprotein, low density lipoprotein, chylomicrons and lipoproteins. In *Apob* knockout mice or mice carrying a dysfunctional truncated Apob protein, homozygous embryos at gestational day 9.5 were either runted or appeared to be nonviable, and the few viable homozygous embryos at gestational day 10.5 appeared to be exencephalic. Moreover, even heterozygous mice had significant reductions in total plasma cholesterol and HDL cholesterol compared with wildtype mice [52, 65, 66]. Thus, APOB can have widespread effects and heterozygous allelic variants can reduce biological function. Similar to our results, LEPR rs1137100 G allele showed a significant increase in the risk of an NTD-affected offspring when inherited from the mother (2.43 fold), and an increased risk for lower spina bifida aperta (Lumbar 1 and lower, 3.20 fold) [67]. A study in Ireland of 520 spina bifida cases and their families and 994 controls found over-transmission of the LEPR rs1805134 minor C allele associated with spina bifida (relative risk of 1.5 fold) [68]. Taken together with our data, there is a significant association of genetic variants in lipid metabolism genes and human lumbar sacral spina bifida aperta.

Variants in apoptosis genes are associated with cranial and spinal NTDs, particularly in the frontal encephalocele, spina bifida aperta and spina bifida cystica (Figs. 4 and 5). Variants in both pro-apoptotic (*APAF1, TP53, CASP3* and *CASP9* etc.) and anti-apoptotic genes (*NFKB1*, *IKBKB* etc.) were found in the present study, consistent with the idea that too little or too much cell death can disrupt the fine regulation of NTC. Indeed, mice carrying mutations in apoptosis genes exhibit exencephaly and spina bifida [1, 69] associated with excess neural cells [69]. On the other hand, excessive apoptosis in the lumbar sacral neuroepithelium has been observed with retinoic acid-induced spina bifida [70]. In human NTDs cases, more apoptotic cells were observed in the central nervous tissue [71]. Higher levels of p53 were observed in anencephaly cases [71]. Cleaved caspase 3 levels were elevated in encephalocele cases and cleaved caspase 8 levels were higher in spina bifida cases relative to controls [71]. Even under harsh environment, failure of NTC is probably due to p53 stabilization and excessive apoptosis [72]. Altogether, abnormal apoptosis might be a common causal factor for cranial and spinal NTDs and abnormal apoptosis might contribute to a wide variety of human NTDs.

Adhesion is key process for fusion of the neural folds and genetic disturbance that alters the adhesion of neural folds should prevent NTC [10]. In two frontal encephalocele and occipital cervical thoracic spina bifida aperta cases, two pairs of rare variants in ECM and adhesion genes were found (Fig. 7f). Integrin *Itga6/Itga3* double mutant mice *or Hspg2* mutant embryos exhibit exencephaly [73, 74]. We observed PDRVs in both *ITGA6* and *HSPG2* genes in one case. In a study in American Caucasian simplex lumbar sacral spina bifida aperta families, a nucleotide polymorphism in *NCAM1* gene may influence NTD risk [75]. Similarly, we found variants in *NCAM1* and *CDON* adhesion genes in some cases.

## Conclusion

Through sequencing NTC-related genes we explored associations of PDRVs in signaling pathways to subphenotypes of human NTDs. Our studies add a significant body of data relative to the genetic causes of human NTDs. Our identification of rare variants in a wide range of genes in a relatively large cohort of NTD cases and controls provides robust insight into plausible critical genetic factors that regulate human neurulation. Moreover, our studies contribute new insight into NTD phenotype-genotype relationships in human. These results lay a foundation for a more detailed understanding of human NTC and how variations in genetic factors may lead to NTDs.

## Supplementary information

**Additional file 1: Table S1**. The summary for genomic DNA sequencing targeted genes.

**Additional file 2: Figure S1.** Summary on PDRVs burden genes related functional pathways in other spinal phenotypes. Summary of PDRVs burden genes in cases affected with spinal NTD phenotypes in other segments. Numbers in brackets means occurrences of PDRVs in each gene, numbers or gene symbols in green represent missense PDRVs. Gene symbols linking with colon represents two concurrent PDRVs in case.

## Data Availability

The datasets used and/or analyzed during the current study are available from the corresponding author on reasonable request.
