# Supplementary material for: Association between rare variants in specific functional pathways and human neural tube defects multiple subphenotypes
Source: Neural Dev. 2020 Jul 10;15:8. doi: 10.1186/s13064-020-00145-7 (PMC7353782; doi:10.1186/s13064-020-00145-7)
Supplement: Supplementary file 1 — Additional file 1: Table S1. The summary for genomic DNA sequencing targeted genes. [file 13064_2020_145_MOESM1_ESM.docx]

Supplementary Table 1: The summary for genomic DNA sequencing targeted genes

| **Pathway** | **Coding DNA sequencing length (bp)** | **Genes number** | **Genes** |
| --- | --- | --- | --- |
| ECM and cell adhesion | 70275 | 18 | SNAI2, CDON, CTHRC1, VASP, LAMA5, HSPG2, ITGA6, ITGAM, VEGFA, GJA1, NCAM1, ADAM10, FLRT3, COL18A1, FREM2, EFNA5, EPHA7, GRHL3 |
| Chromatin modification | 66498 | 24 | NAP1L2, ACTL6A, BRD2, CECR2, CREBBP, DNMT3A, DNMT3B, KAT2A, EP300, EZH2, HDAC1, HIRA, JARID2, PRMT1, PRMT2, REST, RYBP, SMARCA4, SMARCC1, SOX1, SUZ12, CITED2, ATRX, TFAP2A |
| Planar cell polarity signaling | 62259 | 20 | CELSR1, CELSR2, CELSR3, DVL1, DVL2, DVL3, FUZ, FZD3, FZD6, PRICKLE1, PRICKLE2, WNT11, RHO, VANGL1, VANGL2, SMURF1, SMURF2, RHOA, PTK7, SCRIB |
| One carbon metabolism | 57387 | 32 | AHCY, ALDH1L1, AMD1, BHMT, BHMT2, CBS, CTH, CUBN, DHFR, FOLH1, FOLR1, FOLR2, FOLR3, FTCD, GART, MAT1A, MAT2A, MTHFD1, MTHFR, MTR, MTRR, PCMT1, SARDH, GAMT, SHMT1, SLC19A1, TCN2, TRDMT1, TYMS, MUT, NAT1, UCP2 |
| Cytoskeleton | 40536 | 21 | ABI1, ABI2, ARHGAP35, CFL1, COBL, ENAH, PALLD, SHROOM1, SHROOM2, SHROOM3, SHROOM4, GNA13, VCL, MARCKSL1, MARCKS, PHACTR4, FGF2, FGF21, FGF23, FGF8, FGF9 |
| Protein processing | 33663 | 8 | NUP133, NUP50, NUP188, NUP85, NUP155, NUP98, HECTD1, TCOF1 |
| Neural development | 33420 | 15 | OPHN1, GFAP, ADNP, ALX1, ATOH1, DLX2, FOXN1, FOXP4, GCM1, NPAS3, ZIC1, ZIC2, ZIC3, NES, TRPM6 |
| Glucose metabolism | 26970 | 15 | POMT1, FOXO1, TSC1, PEMT, GAPDH, HK1, INS, INSR, CHKA, SRD5A3, HK2, SLC2A4, PAG1, STK11, SLC2A1 |
| MAPK signaling | 24741 | 14 | MAP3K4, MAPK8, MAPK8IP1, MAPK9, MEF2C, NF1, TAB2, FAM48A, CXCR4, PRKCG, PRKCB, PRKACB, PRKACA, PDGFRA |
| Canonical Wnt signaling and Wnt-Ca^2+^ signaling | 22818 | 11 | FRZB, FZD4, FZD5, FZD7, FZD8, FZD9, LRP6, CTNNB1, WNT5A, NFATC3, NFATC4 |
| Cilia | 22698 | 7 | BBS4, CEP290, DNAAF1, IFT172, INTU, MKS1, IQCB1 |
| DNA repair and damage | 21778 | 12 | ERCC2, XRCC1, XRCC3, APEX1, OGG1, MGMT, RFC1, BRCA1, GADD45A, HIPK1, HIPK2, TERC |
| Notch signaling | 20973 | 10 | TWIST1, MIB2, HES1, HES3, NOTCH3, NUMB, PSEN1, JAG1, PSEN2, NTN1 |
| Apoptosis | 19560 | 14 | APAF1, CASP3, CASP9, CHUK, CRLF1, CRLF2, FKBP8, TRAF4, TP53, PPP3R1, PDGFC, NFKB1, IKBKB, BCL10 |
| Lipid metabolism | 17694 | 3 | APOB, LEPR, LEP |
| Hedgehog signaling | 15441 | 9 | GAS1, GLI2, PTCH1, RAB23, SHH, SUFU, GPR161, AFP, STIL |
| TGF-beta signaling | 12522 | 8 | GDF1, FKBP1A, BMP1, BMP2, BMP4, NOG, SKI, ZEB2 |
| Homeobox related | 9784 | 10 | HMX3, HOXA1, LHX4, MSX1, MSX2, PAX1, PAX3, PAX7, POU5F1, SIX3 |
| Retinoid metabolism | 6603 | 6 | ALDH1A2, CRABP1, CRABP2, CYP26A1, CYP26B1, RARA |
| Inositol metabolism | 6393 | 4 | INPP5E, ITPK1, TULP3, PIP5K1C |
| Cell cycle | 3669 | 2 | OFD1, GMNN |
| Others | 23433 | 17 | AMBRA1, PRCP, RNMT, TXN2, SNRK, SLC25A19, FLI1 , SP8, LUZP1, T, TBX1, TBX20, CAT, CXCL12, PTGS2, CSK, SOD2 |
