# Supplementary material for: Association between rare variants in specific functional pathways and human neural tube defects multiple subphenotypes
Source: Neural Dev. 2020 Jul 10;15:8. doi: 10.1186/s13064-020-00145-7 (PMC7353782; doi:10.1186/s13064-020-00145-7)
Supplement: Supplementary file 2 — Additional file 2: Figure S1. Summary on PDRVs burden genes related functional pathways in other spinal phenotypes. Summary of PDRVs burden genes in cases affected with spinal NTD phenotypes in other segments. Numbers in brackets means occurrences of PDRVs in each gene, numbers or gene symbols in green represent missense PDRVs. Gene symbols linking with colon represents two concurrent PDRVs in case. [file 13064_2020_145_MOESM2_ESM.pdf]

A
Thoracic lumbar
spina bifida aperta

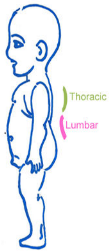

**Total number of cases affected with only Thoracic lumbar spina bifida aperta**	**11**
Associated pathway	Lipid metabolism
Occurences in NTDs *vs.* in control	3/11 *vs.* 10/225
P value	0.029
Burden Gene(s)	*LEPR*(1), *APOB:APOB*(1)

B
Thoracic
spina bifida aperta

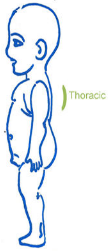

**Total number of cases affected with only Thoracic spina bifida aperta**	**2**
Associated pathway	Protein processing
Occurences in NTDs *vs.* in control	2/2 *vs.* 19/225
P value	0.037
Burden Gene(s)	*NUP98:NUP188*(1)

C
Thoracic lumbar
spina bifida occulta

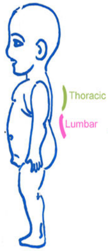

**Total number of cases affected with only Thoracic lumbar spina bifida occulta**	**5**
Associated pathway	Chromatin modification
Occurences in NTDs *vs.* in control	4/5 *vs.* 27/225
P value	0.013
Burden Gene(s)	*CECR2*(2), *PRMT1*(1), *HIRA*(1)
Associated pathway	Neural development
Occurences in NTDs *vs.* in control	3/5 *vs.* 12/225
P value	0.008
Burden Gene(s)	*ADNP*(1), *TRPM6*(2)

D
Lumbar
spina bifida cystica

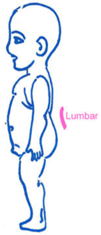

**Total number of cases affected with only Lumbar spina bifida cystica**	**3**
Associated pathway	Retinoid metabolism
Occurences in NTDs *vs.* in control	1/3 *vs.* 0/225
P value	0.017
Burden Gene(s)	*CYP26B1*

E
Thoracic
spina bifida cystia

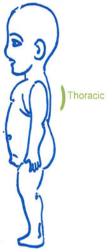

**Total number of cases affected with only Thoracic spina bifida cystica**	**1**
Associated pathway	Apoptosis
Occurences in NTDs *vs.* in control	1/1 *vs.* 4/225
P value	0.042
Burden Gene(s)	*CASP9*

Figure S1 Zou et al.
